# *In Vivo* Visualization of Notch1 Proteolysis Reveals the Heterogeneity of Notch1 Signaling Activity in the Mouse Cochlea

**DOI:** 10.1371/journal.pone.0064903

**Published:** 2013-05-31

**Authors:** Zhiyong Liu, Zhenyi Liu, Bradley J. Walters, Thomas Owen, Raphael Kopan, Jian Zuo

**Affiliations:** 1 Department of Developmental Neurobiology, St. Jude Children’s Research Hospital, Memphis, Tennessee, United States of America; 2 Integrated Program in Biomedical Sciences, University of Tennessee Health Science Center, Memphis, Tennessee, United States of America; 3 Department of Developmental Biology, Washington University School of Medicine, St. Louis, Missouri, United States of America; 4 Division of Dermatology, Washington University School of Medicine, St. Louis, Missouri, United States of America; 5 University of Bath, Bath, United Kingdom; Texas A&M University, United States of America

## Abstract

Mechanosensory hair cells (HCs) and surrounding supporting cells (SCs) in the mouse cochlea are important for hearing and are derived from the same prosensory progenitors. Notch1 signaling plays dual but contrasting and age-dependent roles in mouse cochlear development: early lateral induction and subsequent lateral inhibition. However, it has been difficult to directly visualize mouse cochlear cells experiencing various levels of Notch1 activity at single cell resolution. Here, we characterized two knock-in mouse lines, *Notch1^Cre (Low)/+^* and *Notch1^Cre (High)/+^*, with different Cre recombinase activities, that can detect Notch1 receptor proteolysis or Notch1 activity at high and low thresholds, respectively. Using both lines together with a highly sensitive Cre reporter line, we showed that Notch1 activity is nearly undetectable during lateral induction but increases to medium and high levels during lateral inhibition. Furthermore, we found that within the neonatal organ of Corti, the vast majority of cells that experience Notch1 activity were SCs not HCs, suggesting that HCs kept undetectable Notch1 activity during the entire lineage development. Furthermore, among SC subtypes, ∼85–99% of Deiters’ and outer pillar cells but only ∼19–38% of inner pillar cells experience medium and high levels of Notch1 activity. Our results demonstrate that Notch1 activity is highly heterogeneous: 1) between lateral induction and inhibition; 2) between HC and SC lineages; 3) among different SC subtypes; 4) among different cells within each SC subtype. Such heterogeneity should elucidate how the development of the cochclear sensory epithelium is precisely controlled and how HC regeneration can be best achieved in postnatal cochleae.

## Introduction

Sound detection in the mammalian inner ear is mediated via mechanosensory hair cells (HCs) in the sensory epithelium of the cochlea, also referred to as the organ of Corti [1]–[4]. The organ of Corti contains three rows of outer hair cells (OHCs) and one row of inner hair cells (IHCs), which are surrounded by different types of supporting cells (SCs): inner pillar cells (IPCs), outer pillar cells (OPCs) and Deiters’ cells (DCs) [1]. As demonstrated by linage tracing in the mouse cochlea [5]–[7], HCs and SCs are derived from the same prosensory progenitor cells. In mouse cochlear development, the period between embryonic day (E) 11 and E14 is defined as the early prosensory phase [8]–[10], when the lateral induction effects of Notch signaling specify prosensory progenitors [8], [11]–[17]. The period between E14 and perinatal ages is the lateral inhibition phase, when prosensory progenitors undergo differentiation and Notch signaling promotes SC’s, but antagonizes HC’s, fate commitment and differentiation [18], [19].

It remains unknown how Notch signaling evokes such dual but contrasting effects in the development of the inner ear and how cochlear cells sense and respond appropriately to Notch signaling at different developmental stages. Interestingly, Notch signaling also elicits similar contrasting responses in the development of the central nervous system [20] and the pancreas [21] and, in these tissues, Notch signaling influences cells in a level-dependent manner, where low levels of Notch promote cell proliferation and high levels induce quiescence and cell differentiation. Therefore, we hypothesize that Notch activity is relatively low during lateral induction and increases during lateral inhibition in the developing organ of Corti.

While Notch1 is the primary, active Notch receptor during mouse inner ear development [17], it has been challenging to visualize Notch1 activity levels at single cell resolution. Different levels of Notch1 activity have been inferred by the expression levels of downstream target genes (e.g., *Jagged1* and *Hes* family genes) [22]–[24], or their recapitulation in reporter mice (Hes5-GFP) [25], but these methods have limitations [26]. For example, Jagged1 and Hey2 also respond to input from Wnt signaling [24], [27] and fibroblast growth factor (FGF) signaling [28]. To overcome such limitations and to visualize Notch1 signaling more directly, we utilized a genetic lineage tracing approach involving *Notch1^Cre (Low)/+^* and *Notch1^Cre (High)/+^* mouse strains [26], [29].

**Figure 1:**
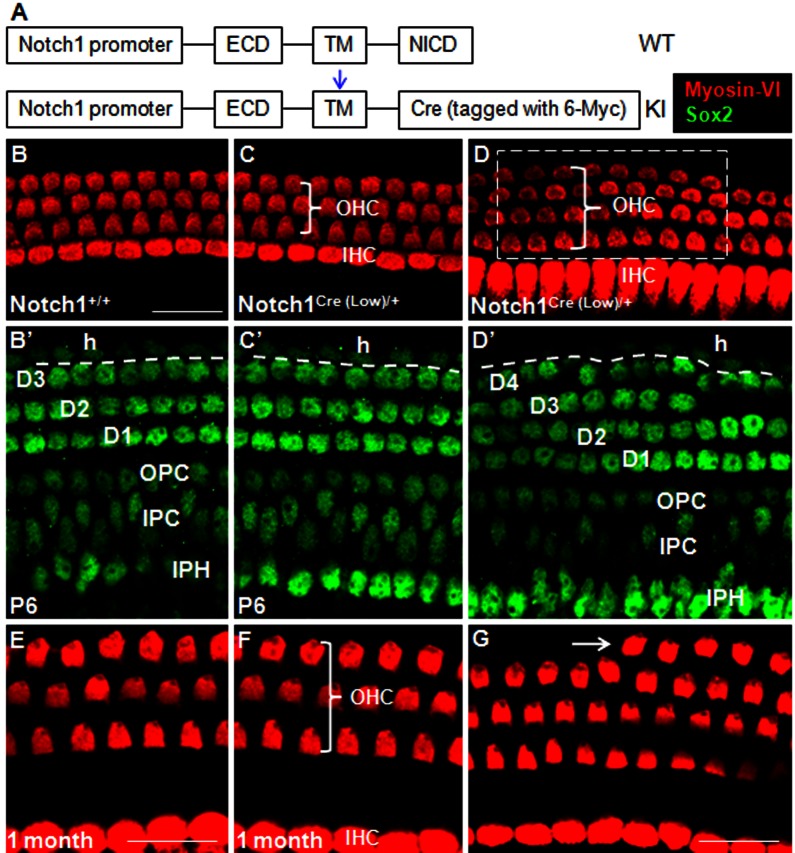
Characterization of the *Notch1* *^Cre (Low)/+^* mouse line. (**A**) Schematic illustration of *Notch1^Cre/+^* mice. The NICD was replaced by 6×Myc–tagged Cre recombinase. The blue arrow represents the cleavage site. (**B–D’**) Comparison between cochleae from *Notch1^Cre/+^* mice and control mice (*Notch1^+/+^*). (B, B’) Myosin-VI+ OHCs (three rows; red) and IHCs (one row; red) sit above Sox2+ SCs (green) in a control (*Notch1^+/+^*) cochlea at P6. (C, C’) In most regions across the entire cochlea, *Notch1^Cre (Low)/+^* mice is indistinguishable from controls. (D, D’) An extra row of DCs always appear underneath the fourth row of OHCs (white dotted rectangular area) in *Notch1^Cre (Low)/+^* cochleae. Although extra DCs and OHCs are frequently observed, each of them spans only a short stretch. The Sox2+ cells outside the dotted line (B’, C’, and D’) are Hensen cells (h). (**E–G**) Morphology of HCs at P30 in control (E) and *Notch1^Cre (Low)/+^* mice (F–G). The distance between OHCs and IHCs is extended. The extra row of OHCs (arrow in G) in *Notch1^Cre (Low)/+^* mice survive and align well with surrounding HCs. D1–D4: three or four rows of Deiters’ cell; OPC: outer pillar cell; IPC: inner pillar cell; IPH: inner phalangeal cell; h: Hensen’s cell. ECD: extracellular domain; TM: transmembrane domain; NICD: Notch1 intracellular domain. Bars: 20 µm. Bar in (B) also applies to C–D’. Bar in (E) also applies to (F).

In both *Notch1^Cre (Low)/+^* and *Notch1^Cre (High)/+^* lines, Notch1 intracellular domain (NICD) was replaced by Cre recombinase with the nuclear localization signal (NLS), resulting in a null mutation of Notch1 (Fig. 1A). The differences between the two lines are mainly two-fold: first, Cre is tagged with 6×Myc in the *Notch1^Cre (Low)/+^* line, while Cre is not tagged with 6×Myc in the *Notch1^Cre (High)/+^* line; second, an extra copy of SV40 polyadenylation signal is added to the end of the construct in *Notch1^Cre (High)/+^* line, which increase the *Notch1/Cre* mRNA level by two folds [29], [30]. These improvements make the Cre-mediated lineage tracing in *Notch1^Cre (High)/+^* line much more sensitive (or of a lower detecting threshold) than in the *Notch1^Cre (Low)/+^* line. For example, in lung tissue, only cells having high levels of Notch1 activity can be traced in the *Notch1^Cre (Low)/+^* line, whereas in the *Notch1^Cre (High)/+^* line, cells with both high and medium levels of Notch1 activity can be traced [31]. In addition, when combined with a floxed Notch1 allele (*Notch1^Cre (Low)/flox^* or *Notch1^Cre (High)/flox^*), *Notch1^Cre (High)^* achieves very high level of self-excision in embryonic endothelial cells, causing embryos to die at around E10.5; in contrast, the self-excision efficiency of *Notch1^Cre (Low)^* is so low that the embryos develop normally and the pups could be born at expected Mendelian ratios [29]. Last, heterozygous mice of both *Notch1^Cre (Low)/+^ and Notch1^Cre (High)/+^* lines are fertile and viable whereas homozygous mice die at ∼E9.5, consistent with two independent *Notch1^−/−^* mouse lines previously characterized [32], [33].

Both *Notch1^Cre (Low)/+^* and *Notch1^Cre (High)/+^*-mediated lineage tracing recapitulates Notch1 proteolysis, but at different levels of sensitivity. When Notch1 activity is sufficiently low, Cre activity remains undetectable in either mouse line; when Notch1 activity is high, Cre activity in both lines are activated; and when Notch1 activity is at an intermediate level, the *Notch1^Cre (High)/+^* line exhibits detectable Cre activity while the *Notch1^Cre (Low)/+^* line does not. These Cre activities are readily visualized by crossing *Notch1^Cre/+^* mice with floxed-stop reporter lines. Our lineage tracing results reported here support the hypothesis that Notch1 activity differs among different cochlear cell types, and between stages of lateral induction and lateral inhibition. Thus these *Notch1^Cre/+^* lines can be widely used in other systems to ascertain variable levels of Notch1 activity *in vivo*.

## Results

### Characterization of Both *Notch1^Cre (Low)/+^ and Notch1^Cre (High)/+^* Alleles in the Mouse Cochlear Development

We first described and characterized the *Notch1^Cre (Low)/+^* and *Notch1^Cre (High)/+^* lines.

Heterozygous mice of both lines exhibited identical minor phenotypes of haploinsuffiency in the organs of Corti, thus we present here data only from the *Notch1^Cre (Low)/+^* mice (Fig. 1A). Like the control wild-type littermates (*Notch1^+/+^*), *Notch1^Cre (Low)/+^* mice had 3 predominant rows of OHCs and 1 row of IHCs at postnatal day (P) 6 (Fig. 1B and C). However, there were discontinuous patches distributed along the length of the cochlear duct in which a 4^th^ row of OHC was observed (white rectangular area in Fig. 1D). Interestingly, extra Sox2+ SCs were also found in the same confocal scanning area where ectopic OHCs were present at P6 (Fig. 1B’–D’; *n* = 3). These extra HCs and SCs survived at adult ages (Fig. 1E–G). Furthermore, whole-mount analysis showed that there was no substantial difference in length of the entire cochlear duct between *Notch1^+/+^* (6050 µm ±110 µm) and *Notch1^Cre (Low)/+^* (6160 µm ±191 µm) mice (*n* = 3 in each group), which rules out the possibility that the increased density of HCs or SCs in *Notch1^Cre (Low)/+^* mice are secondary phenotypes arising from a shortened cochlear duct. Such a phenotype is consistent with presence of supernumerary SCs in the *Hes1/Hes5/Hey1* or *Hes1/Hes5/Hey2* compound mutant mice [22], [23] and the *Notch1* ^+/−^ mice [34].

### Heterogeneity of Notch1 Activity Levels between Lateral Induction and Inhibition Stages of Cochlear Development

Notch1 is turned on at the onset of inner ear development, and the Jagged1 is the major Notch1 ligand in lateral induction stage [9], [11], [24], [27]. The strength of NICD immunostaining at lateral induction is much weaker than that of lateral inhibition stage [35], [36]. Because severe phenotypes were observed in cochleae where Notch1 activity is lost during lateral induction stage [12], [13], we asked whether an alternative way is available to better detect Notch activities in cochlear cells at lateral induction stage. We opted to use Cre-mediate lineage tracing which identifies all cells that have experienced Notch activity at single cell resolution, irrespective of their temporal and spatial characteristics.

**Figure 2:**
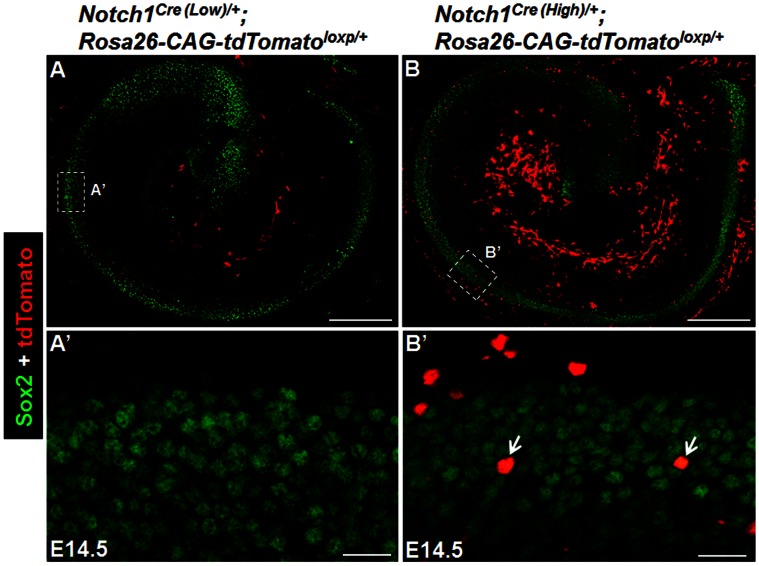
*Notch1^Cre/+^*-mediated reporter expression is difficult to detect in the cochlear prosensory domain at embryonic day (E) 14.5. (A–A’) A single slice of confocal image demonstrating that tdTomato reporter expression (red) was undetectable in Sox2 positive (green) sensory precursor cells in cochleae of *Notch1^Cre (low)/+^; Rosa26-CAG-tdTomato^loxp/+^* mice at E14.5. (A’) is the high magnification image of the rectangular region in (A) taken in the organ of Corti region. (B–B’) A single slice of confocal image taken in cochleae of *Notch1^Cre (High)/+^; Rosa26-CAG-tdTomato^loxp/+^*mice. (B’) is the high magnification image of the rectangular region in (B) taken in the organ of Corti region, showing that a few cells were Sox2+/tdTomato+ (arrows), whereas the majority were Sox2+ only. Scale bar is 200 µm (A, B), 20 µm (A’, B’).

We crossed the *Notch1^Cre (Low)/+^ and Notch1^Cre (High)/+^* lines with a highly sensitive *Rosa26-CAG-tdTomato^ loxp/+^* reporter line which would express tdTomato upon floxed STOP excision by Cre liberated from cell membrane after the mimics of Notch1 proteolysis [37]. Thus tdTomato labels cells that have experienced Notch activities at any point in their lineage. By E14.5, no tdTomato+ cell was observed inside the organ of Corti of *Notch1^Cre (Low)/+^; Rosa26-CAG-tdTomato^ loxp/+^* mice (Fig. 2A–A’). However, a small number (0.97% ±0.3%) of the Sox2+ cells in the cochlear prosensory regions were tdTomato+ in *Notch1^Cre (High)/+^; Rosa26-CAG-tdTomato^ loxp/+^* mice (Fig. 2B–B’). Together, consistent with the NICD immunostaining approach [36], these support that Notch1 activity is generally undetectable or very low but not completely absent in the lateral induction period.

### Heterogeneity of Notch1 Activities Across Cell Types during the Lateral Inhibition Stage of Cochlear Development

We next determined cochlear cell types experiencing Notch1 activity during lateral inhibition stage in two genetic models: *Notch1^Cre (High)/+^*; *Rosa26-CAG-tdTomato^loxp/+^* and *Notch1^Cre (Low)/+^; Rosa26-CAG-tdTomato^loxp/+^*. We analyzed the reporter tdTomato expression at P6 when cochlear cell fate commitment should be completed and Notch1 activity should be diminished, as evidenced by decreased NICD expression during the first postnatal week [36] and the fact that the cochlear SCs become much less responsive to Notch1 inactivation as they age [38]. Furthermore, in the cochleae of *Notch1^Cre (Low)/+^;Rosa26-EYFP^loxp/+^* at E14.5, E16.5, E18.5, P2, and P6, very few EYFP+ SCs began to appear at E18.5 and the number of EYFP+ SCs continuously increased between E18.5 and P6, but stopped further increase after P6 (data not shown). Thus, by P6, all cells experiencing different levels of Notch1 activity during both lateral induction and inhibition in development should be labeled.

**Figure 3:**
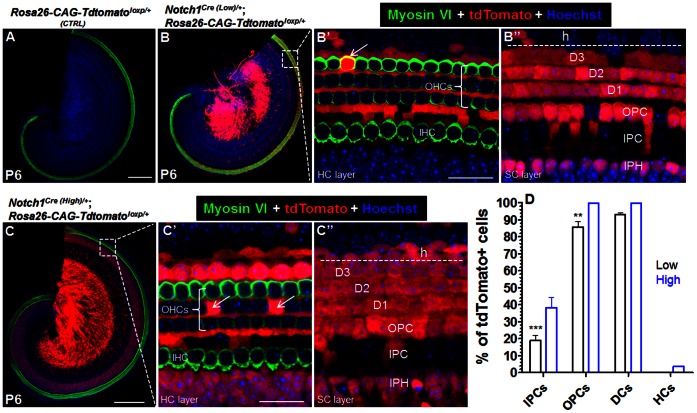
Heterogeneity of Notch activities among different cells. (**A**) Tdtomato expression was absent in control *Rosa26-CAG-tdTomato^loxp/+^* mice. (**B–B”**) Cre-mediated Tdtomato expression in *Notch1^Cre (low)/+^; Rosa26-CAG-tdTomato^loxp/+^* mice. Arrow pointed to a tdTomato+ HC (B’). (**C–C”**) Broader Cre-mediated Tdtomato expression in *Notch1^Cre (High)/+^; Rosa26-CAG-tdTomato^loxp/+^*mice. Two arrows were two tdTomato+ HCs. (**D**) Quantification of tdTomato+cells. The percentage of tdTomato+ HCs in “Low” model was too small to see in the graph. The SEM bar (OPC and DC) was also too small to see in the “High” model. “Low” represents *Notch1^Cre (low)/+^; Rosa26-CAG-tdTomato^loxp/+^*, while “High” does *Notch1^Cre (High)/+^; Rosa26-CAG-tdTomato^loxp/+^*mice. ***p<0.001, **p<0.01. D1-D3: three rows of Deiters’ cell; OPC: outer pillar cell; IPC: inner pillar cell; IPH: inner phalangeal cell; h: Hensen’s cell. Bars: 200 µm (A, C); 20 µm (B’, C’).

In control *Rosa26-CAG-tdTomato^loxp/+^* mice (*n* = 3), *tdTomato* expression was never observed (Fig. 3A). In *Notch1^Cre (Low)/+^; Rosa26-CAG-tdTomato^loxp/+^*mice at P6 (*n* = 4), inside the organ of Corti, many tdTomato+ cells were observed (Fig. 3B–B” and D). For each SC subtype, 19.0% ±3.1% of IPCs, 85.7% ±3.4% of OPCs and 93.0% ±1.0% of DCs were tdTomato+. In contrast, only 0.13% ±0.06% of HCs were tdTomato+ (Fig. 3B’ and D).

In *Notch1^Cre (High)/+^*; *Rosa26-CAG-tdTomato^loxp/+^* at P6 (*n* = 4), similar observations with higher percentages were found (Fig. 3C–C”). Approximately 38.4% ±5.8% of IPCs, 99.4% ±0.4% of OPCs, and 99.8% ±0.2% of DCs were tdTomato+. Again, only 3.5% ±0.9% of total HCs were tdTomato+ (Fig. 3C’ and D). Thus, the absolute percentage differences of tdTomato+cells between *Notch1^Cre (High)/+^; Rosa26-CAG-tdTomato^loxp/+^* and *Notch1^Cre (Low)/+^; Rosa26-CAG-tdTomato^loxp/+^* line are 19.4%, 13.7%, 6.8% and 3.37% for IPCs, OPCs, DCs and HCs, respectively. To highlight the difference, we normalized the percentage to each other and found that the percentages of tdTomato+cells in *Notch1^Cre (Low)/+^; Rosa26-CAG-tdTomato^loxp/+^* are 49.5%, 86.2%, 93.0% and 3.7% of those of *Notch1^Cre (High)/+^; Rosa26-CAG-tdTomato^loxp/+^* for IPCs, OPCs, DCs and HCs, respectively. Taken together, the data strongly suggest heterogeneous Notch1 activity among various cell types within the organ of Corti at lateral inhibition stage and a distinct difference in the sensitivity of the two *Notch1^Cre/+^* mouse lines to different levels of Notch activity.

In *Notch1^Cre (High)/+^*; *Rosa26-CAG-tdTomato^loxp/+^* and *Notch1^Cre (Low)/+^; Rosa26-CAG-tdTomato^loxp/+^* mice, the percentages of tdTomato+ cells at P6 were similar to those analyzed at P21 (data not shown) and tdTomato expression was also found in cells in the spiral ganglion region, greater epithelial ridge (GER) cells, inner phalangeal cells (IPhs), Hensen’s cells, Claudius cells and the vessel endothelial cells underneath the basilar membrane (Fig. 3B and C); however these are beyond the focus of our current study.

## Discussion

Our *in vivo* lineage tracing results reported here demonstrate that, during mouse cochlear development, Notch1 activity is heterogeneous in four aspects: 1) between lateral induction and inhibition stages; 2) between HC and SC lineages; 3) among different SC subtypes; and 4) among different cells within each SC subtype.

The genetic approach of *Notch1^Cre/+^* -mediated lineage tracing is reliable to reflect the Notch activity that cells experienced during development. In support, in a previous study, retinoic acid (RA) response-element (RARE)-driven Cre mice (*RARE-Cre+*) are used to trace cells experiencing different levels of RA activity. In *RARE-Cre+*; *Rosa26-lacZ^loxp/+^* mice, the posterior but not anterior otocyst cells are X-gal+ [39]. These findings are consistent with the fact that a lower level and brief RA signaling activity is present at the anterior side of the otocyst, while a higher and longer-lasting RA activity at the posterior end [40].

In our two *Notch1^Cre/+^* models (*Notch1^Cre (High)/+^* and *Notch1^Cre (Low)/+^*), the readout of *tdTomato* reporter expression is primarily dependent on the dosage of Cre activity within each individual cell which is proportional to the level of Notch1 signaling each cell is experiencing. The recombinase Cre level/activity in *Notch1^Cre (High)/+^* was reported to be much higher than in *Notch1^Cre (Low)/+^* [29], [31]. We therefore defined that: 1) those cells without tdTomato expression in either *Notch1^Cre (High)/+^; Rosa26-CAG-tdTomato^loxp/+^* or *Notch1^Cre (low)/+^; Rosa26-CAG-tdTomato^loxp/+^* were cells with low to undetectable Notch1 activities; 2) those cells with tdTomato expression in *Notch1^Cre (High)/+^; Rosa26-CAG-tdTomato^loxp/+^* but not in *Notch1^Cre (low)/+^; Rosa26-CAG-tdTomato^loxp/+^* were those with medium Notch1 activities; and 3) those cells with tdTomato expression in both *Notch1^Cre (low)/+^; Rosa26-CAG-tdTomato^loxp/+^* and *Notch1^Cre (High)/+^; Rosa26-CAG-tdTomato^loxp/+^* experienced high Notch1 activities. In support, our results demonstrated that Notch1 activity is generally low except a few cells during lateral induction (by E14.5); but it dramatically increases to medium and high levels in many cells by P6 during lateral inhibition. These results are consistent with NICD immunostaining and other loss-of-function genetic studies of Notch1 signaling [36], [41], and further validate our *Notch1^Cre/+^* lineage tracing approach. Finally, we found that *Notch1^Cre (High)/+^; Rosa26-CAG-tdTomato^loxp/+^* and *Notch1^Cre (low)/+^; Rosa26-CAG-tdTomato^loxp/+^* cochleae at P21 exhibited similar reporter expression patterns as those at P6; these results are consistent with previous results that Notch1 levels decrease with age, such as the down-regulation of *Hes5* expression in *Hes5-EGFP+* transgenic mice [25], the decrease in NICD staining in older SCs [36], and the declining responsiveness of SCs to modulations of Notch1 activity [38].

Using this *in vivo* lineage tracing strategy, we observed several interesting findings at P6 cochleae during lateral inhibition. First, 96.5% of HCs had low, while only 3.37% had medium and 0.13% had high levels of Notch1 activities. These HCs with detectable levels of Notch1 activity might have been, in part, newly converted from SCs at late embryonic ages, because of the haploinsufficiency of Notch1 heterozygous alleles (Fig. 1). Alternatively, they may be original HCs that somehow experienced medium or high Notch1 activities and yet still maintained a HC fate. However, we cannot yet distinguish between these two explanations and both scenarios may contribute to the detected Notch1 activity in HCs. Nonetheless, the Notch1 signaling in neonatal HCs might not necessarily affect their development, as shown in our recent study where ectopic expression of NICD increased Sox2 and Prox1 expression in endogenous HCs without detectable hearing abnormalities [42].

Second, in contrast to HCs, the SC lineage significantly increases Notch1 activity during lateral inhibition stage. In cochleae of *Notch1^Cre (High)/+^*; *Rosa26-CAG-tdTomato^loxp/+^*, very few (∼ 0.97%) progenitor cells were tdTomato+ by E14.5 (Fig. 2B), and only ∼ 3.5% of total HCs were tdTomato+ by P6 (Fig. 3C’ and D). These results support that the common progenitor cells during lateral induction stage must experience low levels of Notch1 activity, otherwise many HCs would be tdTomato +. Recently, two reports have shown that Notch1 is not required to specify or maintain the properties of progenitor cells in the cochlea [35], [43]. One simple explanation might be that the loss of low level of Notch1 during lateral induction is compensated by other signaling pathways such as Wnt and Fgf [24], [44]–[46]. However, during lateral inhibition, medium to high levels of Notch1 activity cannot be simply compensated, a conjecture that is consistent with loss-of-function study of Notch activity during late embryonic or neonatal cochlear development [12], [18], [28], [41].

Third, there appears to be significant heterogeneity of Notch1 activity levels among SCs and even within each of the three subtypes in the organ of Corti. Among DCs, 0.2%, 6.8%, and 93.0% of the cells experienced low, medium, and high levels of Notch1 activity, respectively. Similarly, 0.6%, 13.7%, and 85.7% of OPCs experienced low, medium, and high levels of Notch activity, respectively. Most surprisingly, for IPCs, 61.6%, 19.4%, and 19.0% experienced low, medium, and high levels of Notch1 activity, respectively. Consistently, using NICD antibody, its staining strength in SCs (including IPCs) near the IHCs is much weaker than that in SCs near the OHCs (i.e. DCs) [36]. Such extensive heterogeneity has significant implications for our understanding of sensory epithelium development and regeneration. It may account for the heterogeneous responses of different SCs to ectopic Atoh1 expression where only ∼10% neonatal PCs and DCs were converted to immature HCs upon ectopic Atoh1 expression [47]. It is possible that SCs with high levels of Notch1 activity would inhibit Atoh1-mediated conversion into HCs; that is, given our observed Notch1 heterogeneity among SCs, 93% of DCs, 85.7% of OPCs and 19% of IPCs with high Notch1 activities could not be converted into HCs. Because loss of Notch1 activity in damaged cochleae promoted conversion of SCs into HCs [48], simultaneous inactivation of Notch1 and ectopic Atoh1 overexpression might be synergistic *in vivo*. Because of low levels of Notch1 activity in 61.6% of IPCs and the proximity of IPCs to Fgf8-expressing IHCs, we speculate that Fgf8/Fgfr3-mediated Fgfr signaling is also critically important and may collaborate with low levels of Notch1 to control IPC development [28], [49]. Such interactions might also explain the fact that only IPCs, but not OPCs and DCs, proliferate when the Sox2 gene is conditionally deleted at neonatal ages [50]. As Notch1 is also important in keeping SCs quiescent at perinatal ages [41], we conjecture that medium or high levels of Notch1 activity can compensate for Sox2 deletion in 99.4% OPCs and 99.8% DCs (and only 38.4% IPCs), preventing their proliferation.

Finally, these two new *Notch1^Cre/+^* lines exhibit Cre activities at various levels, a valuable tool not only for discriminating levels of Notch1 activity across cell types, tissues, and developmental stages, but also for lineage tracing and genetic manipulation of various genes specifically in cells that experience different levels of Notch1 signaling. In the cochlea, these mouse lines would thus be invaluable for such manipulations in SCs after E16. In summary, our results revealed significant heterogeneity of Notch1 signaling during cochlear development and will have significant implications in our understanding of the development of the organ of Corti and potentially for HC regeneration in mammalian cochleae.

## Materials and Methods

### Mice Strains and Embryonic Age Designation

*Notch1^Cre (Low)/+^* (stock number: 006953) [26] and *Rosa26-CAG-tdTomato^loxp/+^* (stock number: 007908) [51] mice were purchased from The Jackson Laboratory (Bar Harbor, ME). *Notch1^Cre (High)/+^* mice were described in [29]–[31]. Mice were crossed at 5 pm, and checked for presence of the vaginal plug at 7 am the next day. If plugs were present, the morning was designated as E0.5. *Notch1^Cre (Low)/+^; Rosa26-CAG-tdTomato^loxp/+^* mice were bred at St. Jude Children’s Research Hospital (St. Jude). *Notch1^Cre (High)/+^; Rosa26-CAG-tdTomato^loxp/+^* mice were maintained in the animal facility at Washington University, and inner ear samples fixed in 4% paraformaldehyde (PFA) were shipped to and analyzed at St. Jude. All animal work conducted during the course of this study was approved by the Institutional Animal Care and Use Committee at St. Jude and Washington University and performed according to NIH guidelines.

### Histology and Immunofluorescence

Preparation of embryonic, neonatal, and adult-age inner ear samples have been described previously [52]. All samples were examined by using a Zeiss LSM 700 confocal microscope. The following primary antibodies were used: anti-Myosin-VI (rabbit, 1∶200, 25–6791, Proteus Bioscience, Ramona, CA), anti-Sox2 (goat, 1∶1000, sc-17320, Santa Cruz Biotechnology, Santa Cruz, CA). The following secondary antibodies were used: goat anti rabbit Alexa Fluor 568 (1∶1000, A11036, Invitrogen), donkey anti goat Alexa Fluor 568 (1∶1000, A11057, Invitrogen), donkey anti rabbit Alexa Fluor 647 (1∶1000, A31573, Invitrogen).

### Cell Counting

Embryonic and neonatal cochlear samples were divided into two parts, whereas adult samples were divided into three parts. We purposely left a tiny cut in spiral ganglion areas of each turn to help distinguish the two ends under the confocal microscope. With the preliminary low-magnification image, we first measured the length of each turn by drawing a curved line in the middle of OHCs and IHCs and then added up the length of the three turns. Confocal Z stac (40× oiled lens) scanning was performed at 1 µm intervals to tdTomato or Myosin-VI or Sox2-expressing cells, with Hoechst33342 being used to label cell nuclei. This approach was used to reduce the counting variations among different samples. For each SC subtype, the percentage of SCs traced by tdTomato was calculated by normalizing the number of tdTomato+cells with respect to the total number of SCs (using Sox2 as a marker) in the same confocal Z stack scanning area.

### Statistical Analysis

All data were expressed as mean ± S.E.M. Each cell type counting between 2 different genetic models at P6 was compared using a one-way ANOVA followed by a Student’s *t* test with a Bonferroni correction. Statistical analysis was conducted using GraphPad Prism 5.0 Software.

## References

[1] Kelley MW (2006) Regulation of cell fate in the sensory epithelia of the inner ear. Nat Rev Neurosci 7: 837–849. 17053809 10.1038/nrn1987

[2] Bok J, Chang W, Wu DK (2007) Patterning and morphogenesis of the vertebrate inner ear. Int J Dev Biol 51: 521–533. 17891714 10.1387/ijdb.072381jb

[3] Kelly MC, Chen P (2009) Development of form and function in the mammalian cochlea. Curr Opin Neurobiol 19: 395–401. 19683914 10.1016/j.conb.2009.07.010 PMC4158839

[4] Kwan T, White PM, Segil N (2009) Development and regeneration of the inner ear. Ann N Y Acad Sci 1170: 28–33. 19686102 10.1111/j.1749-6632.2009.04484.x PMC7245053

[5] Carroll Driver E, Sillers L, Coate TM, Rose MF, Kelley MW (2013) The Atoh1-lineage gives rise to hair cells and supporting cells within the mammalian cochlea. Dev Biol. 10.1016/j.ydbio.2013.01.005 PMC3652277 23318633

[6] Matei V, Pauley S, Kaing S, Rowitch D, Beisel KW, et al (2005) Smaller inner ear sensory epithelia in Neurog 1 null mice are related to earlier hair cell cycle exit. Dev Dyn 234: 633–650. 16145671 10.1002/dvdy.20551 PMC1343505

[7] Yang H, Xie X, Deng M, Chen X, Gan L (2010) Generation and characterization of Atoh1-Cre knock-in mouse line. Genesis 48: 407–413. 20533400 10.1002/dvg.20633 PMC2885570

[8] Hayashi T, Kokubo H, Hartman BH, Ray CA, Reh TA, et al (2008) Hesr1 and Hesr2 may act as early effectors of Notch signaling in the developing cochlea. Dev Biol 316: 87–99. 18291358 10.1016/j.ydbio.2008.01.006 PMC2362132

[9] Eddison M, Le Roux I, Lewis J (2000) Notch signaling in the development of the inner ear: lessons from Drosophila. Proc Natl Acad Sci U S A 97: 11692–11699. 11050197 10.1073/pnas.97.22.11692 PMC34337

[10] Daudet N, Lewis J (2005) Two contrasting roles for Notch activity in chick inner ear development: specification of prosensory patches and lateral inhibition of hair-cell differentiation. Development 132: 541–551. 15634704 10.1242/dev.01589

[11] Kiernan AE, Ahituv N, Fuchs H, Balling R, Avraham KB, et al (2001) The Notch ligand Jagged1 is required for inner ear sensory development. Proc Natl Acad Sci U S A 98: 3873–3878. 11259677 10.1073/pnas.071496998 PMC31145

[12] Brooker R, Hozumi K, Lewis J (2006) Notch ligands with contrasting functions: Jagged1 and Delta1 in the mouse inner ear. Development 133: 1277–1286. 16495313 10.1242/dev.02284

[13] Kiernan AE, Xu J, Gridley T (2006) The Notch ligand JAG1 is required for sensory progenitor development in the mammalian inner ear. PLoS Genet 2: e4. 16410827 10.1371/journal.pgen.0020004 PMC1326221

[14] Kokubo H, Tomita-Miyagawa S, Hamada Y, Saga Y (2007) Hesr1 and Hesr2 regulate atrioventricular boundary formation in the developing heart through the repression of Tbx2. Development 134: 747–755. 17259303 10.1242/dev.02777

[15] Hartman BH, Reh TA, Bermingham-McDonogh O (2010) Notch signaling specifies prosensory domains via lateral induction in the developing mammalian inner ear. Proc Natl Acad Sci U S A 107: 15792–15797. 20798046 10.1073/pnas.1002827107 PMC2936601

[16] Pan W, Jin Y, Stanger B, Kiernan AE (2010) Notch signaling is required for the generation of hair cells and supporting cells in the mammalian inner ear. Proc Natl Acad Sci U S A 107: 15798–15803. 20733081 10.1073/pnas.1003089107 PMC2936630

[17] Liu Z, Owen T, Fang J, Zuo J (2012) Overactivation of notch1 signaling induces ectopic hair cells in the mouse inner ear in an age-dependent manner. PLoS One 7: e34123. 22448289 10.1371/journal.pone.0034123 PMC3309011

[18] Lanford PJ, Lan Y, Jiang R, Lindsell C, Weinmaster G, et al (1999) Notch signalling pathway mediates hair cell development in mammalian cochlea. Nat Genet 21: 289–292. 10080181 10.1038/6804

[19] Dabdoub A, Puligilla C, Jones JM, Fritzsch B, Cheah KS, et al (2008) Sox2 signaling in prosensory domain specification and subsequent hair cell differentiation in the developing cochlea. Proc Natl Acad Sci U S A 105: 18396–18401. 19011097 10.1073/pnas.0808175105 PMC2587543

[20] Guentchev M, McKay RD (2006) Notch controls proliferation and differentiation of stem cells in a dose-dependent manner. Eur J Neurosci 23: 2289–2296. 16706837 10.1111/j.1460-9568.2006.04766.x

[21] Ninov N, Borius M, Stainier DY (2012) Different levels of Notch signaling regulate quiescence, renewal and differentiation in pancreatic endocrine progenitors. Development 139: 1557–1567. 22492351 10.1242/dev.076000 PMC3317964

[22] Tateya T, Imayoshi I, Tateya I, Ito J, Kageyama R (2011) Cooperative functions of Hes/Hey genes in auditory hair cell and supporting cell development. Dev Biol. 10.1016/j.ydbio.2011.01.038 21300049

[23] Li S, Mark S, Radde-Gallwitz K, Schlisner R, Chin MT, et al (2008) Hey2 functions in parallel with Hes1 and Hes5 for mammalian auditory sensory organ development. BMC Dev Biol 8: 20. 18302773 10.1186/1471-213X-8-20 PMC2277407

[24] Jayasena CS, Ohyama T, Segil N, Groves AK (2008) Notch signaling augments the canonical Wnt pathway to specify the size of the otic placode. Development 135: 2251–2261. 18495817 10.1242/dev.017905 PMC2575054

[25] Hartman BH, Basak O, Nelson BR, Taylor V, Bermingham-McDonogh O, et al (2009) Hes5 expression in the postnatal and adult mouse inner ear and the drug-damaged cochlea. J Assoc Res Otolaryngol 10: 321–340. 19373512 10.1007/s10162-009-0162-2 PMC2757554

[26] Vooijs M, Ong CT, Hadland B, Huppert S, Liu Z, et al (2007) Mapping the consequence of Notch1 proteolysis in vivo with NIP-CRE. Development 134: 535–544. 17215306 10.1242/dev.02733 PMC2583343

[27] Ohyama T, Mohamed OA, Taketo MM, Dufort D, Groves AK (2006) Wnt signals mediate a fate decision between otic placode and epidermis. Development 133: 865–875. 16452098 10.1242/dev.02271

[28] Doetzlhofer A, Basch ML, Ohyama T, Gessler M, Groves AK, et al (2009) Hey2 regulation by FGF provides a Notch-independent mechanism for maintaining pillar cell fate in the organ of Corti. Dev Cell 16: 58–69. 19154718 10.1016/j.devcel.2008.11.008 PMC2696015

[29] Liu Z, Turkoz A, Jackson EN, Corbo JC, Engelbach JA, et al (2011) Notch1 loss of heterozygosity causes vascular tumors and lethal hemorrhage in mice. J Clin Invest 121: 800–808. 21266774 10.1172/JCI43114 PMC3026721

[30] Liu Z, Obenauf AC, Speicher MR, Kopan R (2009) Rapid identification of homologous recombinants and determination of gene copy number with reference/query pyrosequencing (RQPS). Genome Res 19: 2081–2089. 19797679 10.1101/gr.093856.109 PMC2775603

[31] Morimoto M, Liu Z, Cheng HT, Winters N, Bader D, et al (2010) Canonical Notch signaling in the developing lung is required for determination of arterial smooth muscle cells and selection of Clara versus ciliated cell fate. J Cell Sci 123: 213–224. 20048339 10.1242/jcs.058669 PMC2954246

[32] Swiatek PJ, Lindsell CE, del Amo FF, Weinmaster G, Gridley T (1994) Notch1 is essential for postimplantation development in mice. Genes Dev 8: 707–719. 7926761 10.1101/gad.8.6.707

[33] Conlon RA, Reaume AG, Rossant J (1995) Notch1 is required for the coordinate segmentation of somites. Development 121: 1533–1545. 7789282 10.1242/dev.121.5.1533

[34] Zhang N, Martin GV, Kelley MW, Gridley T (2000) A mutation in the Lunatic fringe gene suppresses the effects of a Jagged2 mutation on inner hair cell development in the cochlea. Curr Biol 10: 659–662. 10837254 10.1016/s0960-9822(00)00522-4

[35] Basch ML, Ohyama T, Segil N, Groves AK (2011) Canonical Notch signaling is not necessary for prosensory induction in the mouse cochlea: insights from a conditional mutant of RBPjkappa. J Neurosci 31: 8046–8058. 21632926 10.1523/JNEUROSCI.6671-10.2011 PMC3112354

[36] Murata J, Tokunaga A, Okano H, Kubo T (2006) Mapping of notch activation during cochlear development in mice: implications for determination of prosensory domain and cell fate diversification. J Comp Neurol 497: 502–518. 16736472 10.1002/cne.20997

[37] Madisen L, Zwingman TA, Sunkin SM, Oh SW, Zariwala HA, et al (2010) A robust and high-throughput Cre reporting and characterization system for the whole mouse brain. Nat Neurosci 13: 133–140. 20023653 10.1038/nn.2467 PMC2840225

[38] Groves AK (2010) The challenge of hair cell regeneration. Exp Biol Med (Maywood) 235: 434–446. 20407075 10.1258/ebm.2009.009281 PMC3773238

[39] Dolle P, Fraulob V, Gallego-Llamas J, Vermot J, Niederreither K (2010) Fate of retinoic acid-activated embryonic cell lineages. Dev Dyn 239: 3260–3274. 21046629 10.1002/dvdy.22479

[40] Bok J, Raft S, Kong KA, Koo SK, Drager UC, et al (2011) Transient retinoic acid signaling confers anterior-posterior polarity to the inner ear. Proc Natl Acad Sci U S A 108: 161–166. 21173260 10.1073/pnas.1010547108 PMC3017143

[41] Kiernan AE, Cordes R, Kopan R, Gossler A, Gridley T (2005) The Notch ligands DLL1 and JAG2 act synergistically to regulate hair cell development in the mammalian inner ear. Development 132: 4353–4362. 16141228 10.1242/dev.02002

[42] Liu Z, Owen T, Fang J, Srinivasan RS, Zuo J (2012) In vivo Notch reactivation in differentiating cochlear hair cells induces Sox2 and Prox1 expression but does not disrupt hair cell maturation. Dev Dyn 241: 684–696. 22354878 10.1002/dvdy.23754 PMC3302943

[43] Yamamoto N, Chang W, Kelley MW (2011) Rbpj regulates development of prosensory cells in the mammalian inner ear. Dev Biol 353: 367–379. 21420948 10.1016/j.ydbio.2011.03.016

[44] Hayashi T, Ray CA, Bermingham-McDonogh O (2008) Fgf20 is required for sensory epithelial specification in the developing cochlea. J Neurosci 28: 5991–5999. 18524904 10.1523/JNEUROSCI.1690-08.2008 PMC2597653

[45] Pirvola U, Ylikoski J, Trokovic R, Hebert JM, McConnell SK, et al (2002) FGFR1 is required for the development of the auditory sensory epithelium. Neuron 35: 671–680. 12194867 10.1016/s0896-6273(02)00824-3

[46] Huh SH, Jones J, Warchol ME, Ornitz DM (2012) Differentiation of the lateral compartment of the cochlea requires a temporally restricted FGF20 signal. PLoS Biol 10: e1001231. 22235191 10.1371/journal.pbio.1001231 PMC3250500

[47] Liu Z, Dearman JA, Cox BC, Walters BJ, Zhang L, et al (2012) Age-dependent in vivo conversion of mouse cochlear pillar and deiters’ cells to immature hair cells by atoh1 ectopic expression. J Neurosci 32: 6600–6610. 22573682 10.1523/JNEUROSCI.0818-12.2012 PMC3359704

[48] Mizutari K, Fujioka M, Hosoya M, Bramhall N, Okano HJ, et al (2013) Notch inhibition induces cochlear hair cell regeneration and recovery of hearing after acoustic trauma. Neuron 77: 58–69. 23312516 10.1016/j.neuron.2012.10.032 PMC3573859

[49] Jacques BE, Montcouquiol ME, Layman EM, Lewandoski M, Kelley MW (2007) Fgf8 induces pillar cell fate and regulates cellular patterning in the mammalian cochlea. Development 134: 3021–3029. 17634195 10.1242/dev.02874

[50] Liu Z, Walters BJ, Owen T, Brimble MA, Steigelman KA, et al (2012) Regulation of p27Kip1 by Sox2 maintains quiescence of inner pillar cells in the murine auditory sensory epithelium. J Neurosci 32: 10530–10540. 22855803 10.1523/JNEUROSCI.0686-12.2012 PMC3427024

[51] Srinivas S, Watanabe T, Lin CS, William CM, Tanabe Y, et al (2001) Cre reporter strains produced by targeted insertion of EYFP and ECFP into the ROSA26 locus. BMC Dev Biol 1: 4. 11299042 10.1186/1471-213X-1-4 PMC31338

[52] Liu Z, Owen T, Zhang L, Zuo J (2010) Dynamic expression pattern of Sonic hedgehog in developing cochlear spiral ganglion neurons. Dev Dyn 239: 1674–1683. 20503364 10.1002/dvdy.22302 PMC2963025

